# Twelve-Nitrogen-Atom Cyclic Structure Stabilized by 3*d*-Element Atoms: Quantum Chemical Modeling

**DOI:** 10.3390/ijms23126560

**Published:** 2022-06-12

**Authors:** Oleg V. Mikhailov, Denis V. Chachkov

**Affiliations:** 1 Department of Analytical Chemistry, Certification and Quality Management, Kazan National Research Technological University, K. Marx Street 68, Kazan 420015, Russia; 2 Kazan Department of Joint Supercomputer Center of Russian Academy of Sciences—Branch of Federal Scientific Center “Scientific Research Institute for System Analysis of the RAS”, Lobachevskii Street 2/31, Kazan 420111, Russia; de2005c@gmail.com

**Keywords:** 3*d*-element-nitrogen compound, M(N_12_), molecular structure, DFT quantum chemical calculation method

## Abstract

Using various versions of density functional theory (DFT), DFT M06/TZVP, DFT B3PW91/TZVP, DFT OPBE/TZVP, and, partially, the MP2 method, the possibility of the existence of 3*d*-element (M) compounds with nitrogen having unusual M: nitrogen ratio 1:12, unknown for these elements at the present, was shown. Structural parameter data were presented. It was shown that all MN4 groupings have tetragonal-pyramidal structure. It was noted that the bond lengths formed by nitrogen atoms and an M atom were equal to each other only in the case of M = Ti, V, Cr and Co, whereas for other Ms, they were slightly different; moreover, the bond angles formed by nitrogen atoms and an M atom were equal to 90.0°, or practically did not differ from this value. Thermodynamic parameters, NBO analysis data and HOMO/LUMO images for this compound were also presented. Good agreement between the calculated data obtained using the above three quantum chemical methods was also noted.

## 1. Introduction

In our previous article [1], a quantum chemical calculation of the carbon-nitrogen compound molecular and electronic structures having a structural formula (1) with an unusual ratio between the number of carbon and nitrogen atoms (1:12) was performed, and the principal possibility of its existence was shown using quantum chemical methods DFT B3PW91/TZVP, MP2/TZVP and MP3/TZVP. Owing to the structural formation with the participation of the central carbon atom, the stabilization of the structural fragment of twelve nitrogen atoms (N12) took place. According to the data presented in [2,3,4,5,6,7,8,9,10], this grouping of atoms, if it is capable of existing by itself, is very unstable. It seemed interesting to find out whether the chemical compounds of the general formula (2) C(N12), similar to that described in [1], exist, but contain atoms of various 3*d* elements (M) instead of a carbon atom.(1)
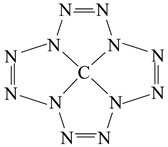

(2)
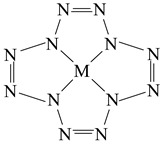


Additionally, in the case of a positive answer to this question, to what degree do the molecular and electronic structure parameters of such compounds, as well as their thermodynamic characteristics, depend on the nature of the M atom? There is no information in the literature about such compounds, although a number of publications were devoted to two-element chemical substances containing atoms of *s*-, *p*- or *d*-elements and nitrogen atoms (in particular, [11,12] and articles by [13,14,15,16,17,18,19,20] in the last 5 years). It should be noted that almost every one of these works mentioned the possible use of such compounds as potential high-energy materials. Consequently, this article will be dedicated to the consideration of chemical compounds having the above formula (2).

## 2. Method

In the given work, the density functional theory (DFT), which combines the standard extended split valence basis set TZVP and the most modern hybrid functional M06 described in [21], was used. For comparison, another version of the DFT method, DFT with the B3PW91 functional, was described extensively in [22,23,24] and used by us, in particular, in [25]. Application of the given version of the DFT method was due to the fact that, according to [22,23,24], it allows one to obtain, as a rule, the most exact (i.e., close to experimental) values of the molecular structures and geometric parameters, as well as much more accurate values for thermodynamic and other physical-chemical parameters in comparison to other DFT method variants. In addition, the molecular and electronic structures of the investigated compounds were calculated using the DFT OPBE/TZVP method, which combines the above-mentioned TZVP basis and the non-hybrid OPBE functional [26,27], that in the case of 3*d*-element complexes gives a fairly accurate ratio of the high-pin state energy stability in regard to the low-spin state and, at the same time, reliably characterizes the key geometric parameters of the metal complexes’ molecular structures [27,28,29,30,31]. As an alternative, the perturbation theory method [32], MP2 [33], in combination with the TZVP basis set was used. These calculations were performed using the Gaussian09 program package [34]. This calculation method was used in our previous article [25], and the correspondence with the found stationary points to the energy minima in all cases was proved by calculating the second derivatives of the energy to the coordinates of the atoms, wherein all equilibrium structures corresponding to the minimum points on the potential energy surfaces had only real (and, moreover, always positive) frequency values. From the optimized structures for further consideration, the one with the lowest total energy was selected. Unfortunately, at the moment we had to limit ourselves to calculations using different versions of the DFT method, because for the analyzed compounds, completing the calculation with any of the higher-level methods (QCISD, CCSD, and even MP3) led us to failure due to the complexity of these methods, as well as our limited time and energy costs. Natural bond orbital (NBO) analysis was performed using NBO version 3.1, integrated with Gaussian09 program package [34] according to the methodology described in [35]. NBO methods are well known for excellent numerical stability and convergence regarding basis set expansion, sensibly proportionate to convergence of energy and other calculated wavefunction properties (unlike Mulliken analysis and related overlap-dependent methods). The standard thermodynamic parameters of formation, *H*^0^_f__,298_, *S*^0^_f__,298_ and *G*^0^_f__,298_ for the M(N_12_) compounds were calculated according to the methodology described in [36].

## 3. Results and Discussion

According to the data from each of the three above-mentioned methods of quantum chemical calculation, most of the M(N_12_) chemical compounds have molecular structures (2) where M–any of 3*d*-elements are capable of independent existence. Sc and Zn are the only exceptions. For the first of them, such a compound cannot arise due to its limited valence capabilities; for the second, although it is possible, it is unlikely (it was confirmed by our calculations). The most important geometric parameters of molecular structures of M(N_12_) compounds for various M (the lengths of chemical bonds between atoms and bond angles) revealed using the DFT M06/TZVP method are presented in Table 1. For comparison, this Table also presents the molecular structure parameters calculated by the same method, for the chemical compound that can be considered as a kind of “progenitor” of the studied metal complexes, namely H_4_(N_12_), as a result of the substitution of all four hydrogen atoms in which all compounds are of type (2). The similar data calculated by the DFT B3PW91/TZVP and DFT OPBE/TZVP methods are presented in Tables S1–S8 (see Supplementary Materials). From these data, all three methods gave very close values for the key parameters of these molecular structure. It seems appropriate to discuss the results produced by any one of them, and namely DFT M06/TZVP as the most advanced among DFT B3PW91/TZVP and DFT OPBE/TZVP methods.

**Figure 1:**
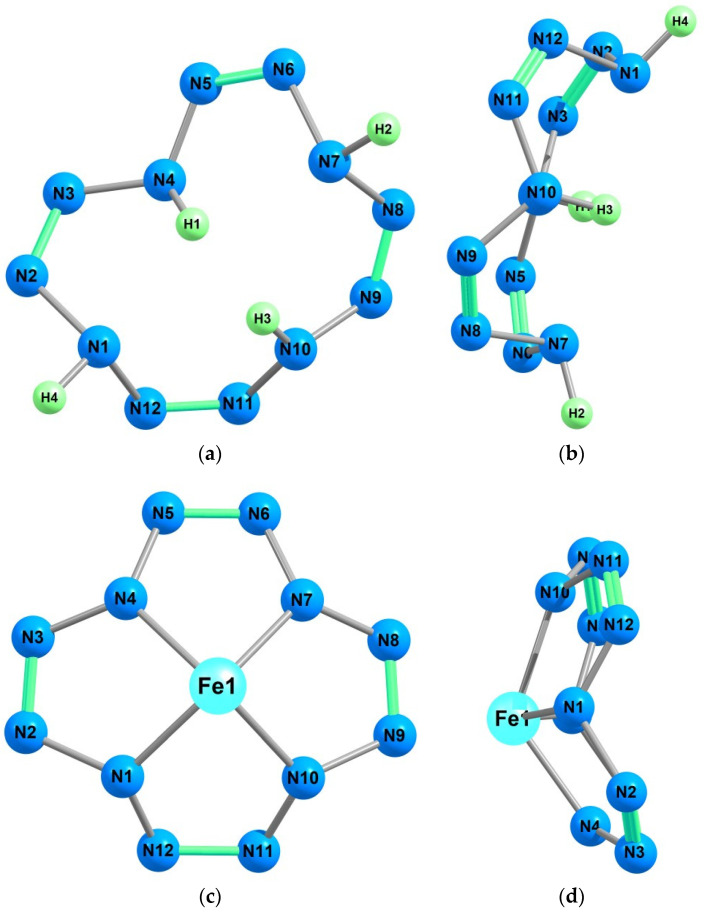

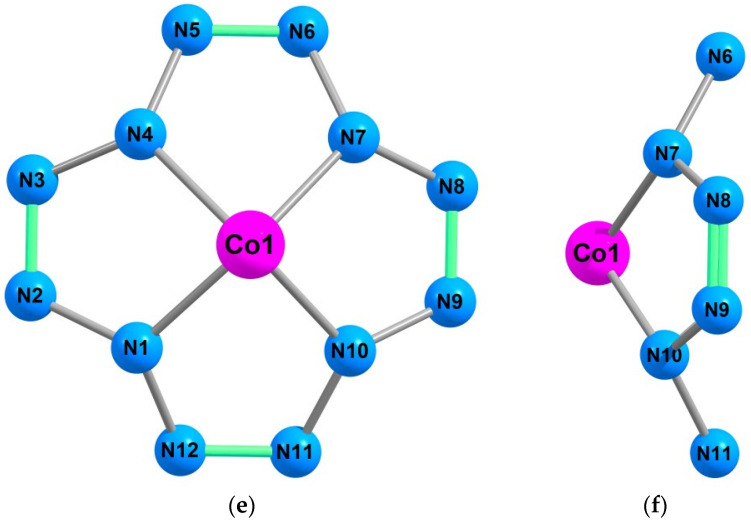
Molecular structures of the compounds H_4_(N_12_) (**a**,**b**), Fe(N_12_) (**c**,**d**) and Co(N_12_) (**e**,**f**) obtained as a result of DFT M06/TZVP quantum chemical calculation: front view (**a**,**c**,**e**), side view (**b**,**d**,**f**).

As can be seen from Table 1, the MN_4_ atom grouping (chelate node) in each of the chemical compounds has a tetragonal-pyramidal structure with a very significant (more than 30°) deviation from the plane formed by four nitrogen atoms bonded to the M atom. Examples of such structures are shown in Figure 1. It should be noted that, in contrast to the grouping of MN_4_ atoms, the grouping of four nitrogen atoms included in the chelate node in all M(N_12_) discussed compounds was strictly flat (the sum of the angles N1N4N7, N4N7N10, N7N10N1 and N10N1N4 in each is 360.0°). Moreover, this grouping had the shape of either a square (in the case of M = Ti, V, Cr, Co) or an isosceles trapezoid (in the case of M = Mn, Fe, Ni, Cu). Accordingly, in the first four complexes, all M–N bond lengths were equal to each other, whereas in the rest, they were not equal (Table 1). It is interesting that the diagonals of these quadrangles (N1—N7) and (N4—N10) in seven of these eight compounds were the same in length and were 325.9 (Ti), 321.9 (V), 326.4 (Cr), 326.4 (Co), 333.4 (Ni) and 343.0 (Cu) pm. For M = Mn, there were small differences between them, 332.9 and 332.7 pm, respectively. Thus, in each of the M(N_12_) considered compounds, the M atom was to some extent elevated above the plane of four donor nitrogen atoms; the height of this rise strongly depended on the M nature and varied from 67.4 pm (in Ni(N_12_)) to 104.4 pm (in Ti(N_12_)) (Table 1), which correlated rather well with the sizes of M(II) and M(IV) of the analyzed 3*d* elements. In contrast to the N4 groups, the 12-membered macrocycles N12 formed by nitrogen atoms were non-coplanar, and very significant deviations from coplanarity took place for them (Table 1). Interestingly, as a result of the formation of any of these compounds, a decrease in the deviation degree from this macrocycle coplanarity compared to that for the H_4_(N_12_) ligand occurred. This deviation, as can be seen from Table 1, varied from 34.7° in the case of Cu(N_12_) to 63.7° in the case of Fe(N_12_) depending on the M nature. Based on this, the M(N_12_) considered compounds could be clearly divided into two equal groups. The first of them (A) included Ti(N_12_), V(N_12_), Cr(N_12_) and Co(N_12_), whose molecular structures have the *C*_4*v*_ symmetry group. The second (B) included Mn(N_12_), Fe(N_12_), Ni(N_12_) and Cu(N_12_), whose molecular structures have the *C*_1_ symmetry group, i.e., devoid of any symmetry elements. The molecular structure of the H_4_(N_12_) ligand was also completely asymmetric. In this structure, two N–H bonds are directed inside the N12 macrocycle, while the other two are outside it (Figure 1). The dipole electric moment values for these compounds calculated using each of the DFT M06/TZVP, DFT B3PW91/TZVP, and DFT OPBE/TZVP methods were very different from zero (Table 2), which is understandable because of the lack of a center of symmetry in each of them. The μ values calculated by two other DFT methods (in Debye units), are given in Table S9 (see Supplementary Materials).

Key data of NBO analysis and, namely, the effective charge values on 3*d*-element atoms and nitrogen atoms for examined chemical compounds obtained by DFT M06/TZVP method, are presented in Table 3. Similar results calculated using the DFT B3PW91/TZVP and DFT OPBE/TZVP methods are presented in Table S10 (see Supplementary Materials). Obviously, numerical values of the squared operator of the angular moment of the total spin of the system <S**2> in the case of Ti(N_12_) and Ni(N_12_) correspond to total spin of system S = 0, in the case of V(N_12_) and Cu(N_12_) – S = 1/2, in the case of Cr(N_12_) and Fe(N_12_) − S = 1, and in the case of Mn(N_12_) and Co(N_12_) – S = 3/2. This allowed us to assume that in the analyzed compounds, in the case of M = Ti, the electronic configuration of the central ion 3*d*^0^ was formed in the cases of M = V – 3*d*^1^, M = Cr − 3*d*^2^, M = Mn – 3*d*^3^, M = Fe – 3*d*^4^, M = Co – 3*d*^5^, M = Ni – 3*d*^6^, and M = Cu – 3*d*^7^ that correspond to the oxidation state of the central M(IV) atom. Two additional points should be noted. Firstly, the effective charges on the M atoms in all cases differed very significantly from the value +4.000 ē that would apply if all chemical bonds between M and N atoms were ionic. Secondly, the charges on both M and nitrogen atoms strongly depended on the atomic nature of the 3*d* element. However, this fact indicates that in the all discussed compounds, a very high degree of delocalization of the electron density was found. The ground states of M(N_12_) are a spin singlet (*M_S_* = 1) (M = Ti, Ni), doublet (M = V, Cu), triplet (M = Cr, Fe) and quartet (M = Mn, Co). In this case, the next excited energy state with a different spin multiplicity *M_S_* (triplet for M = Ti, Ni, quartet for M = V, Cu, singlet for M = Cr, quintet for M = Fe, sextet for M = Mn and doublet for M = Co) was located above the ground state by 125.5; 2.5; 160.7; 63.4; 26.2; 12.5; 58.9; and 27.4 kJ/mol, so that a spin crossover could be expected only for Ni(N_12_).

**Figure 2:**
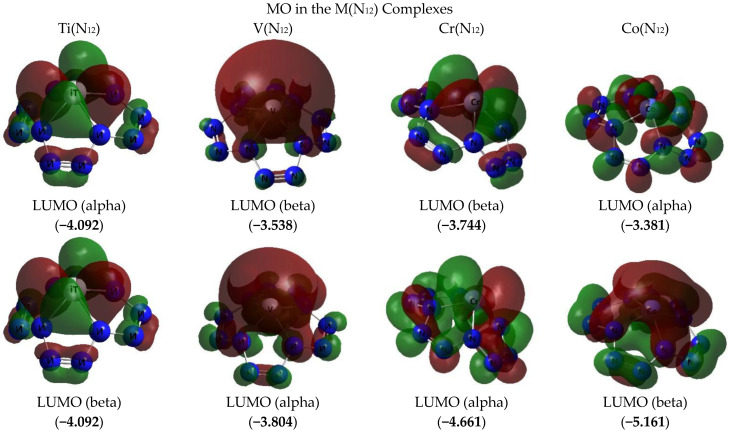

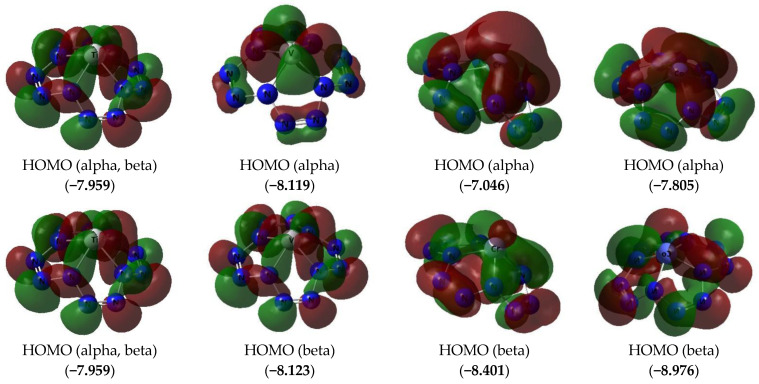
The images of highest occupied (HOMO) and lowest unoccupied (LUMO) molecular orbitals in the M(N_12_)] complexes of group A obtained by DFT M06/TZVP method. The values of energies of these molecular orbitals (in brackets) are given in eV. The symbol “alpha” corresponds to electron with spin (+1/2), “beta”, to electron with spin (−l/2).

**Figure 3:**
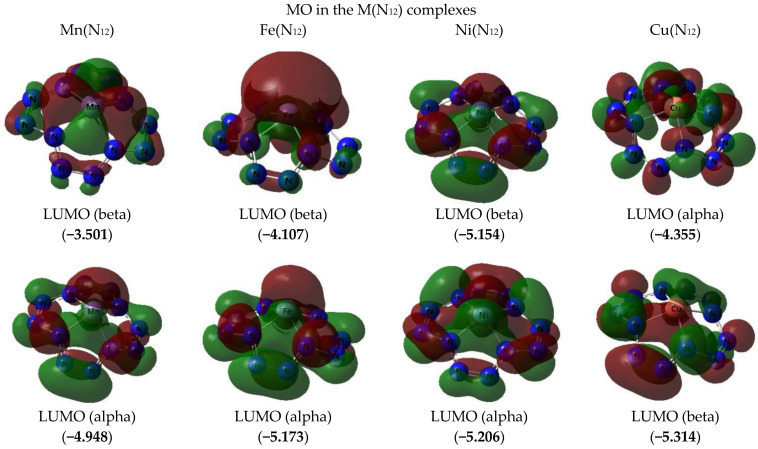

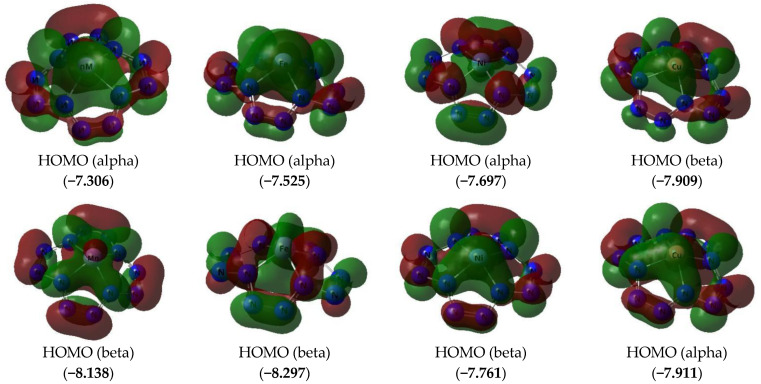
The images of highest occupied (HOMO) and lowest unoccupied (LUMO) molecular orbitals in the M(N_12_)] complex of group B obtained by DFT M06/TZVP method. The values of energies of these molecular orbitals (in brackets) are given in eV. The symbol “alpha” corresponds to electron with spin (+1/2), “beta”, to electron with spin (−l/2).

**Figure 4:**
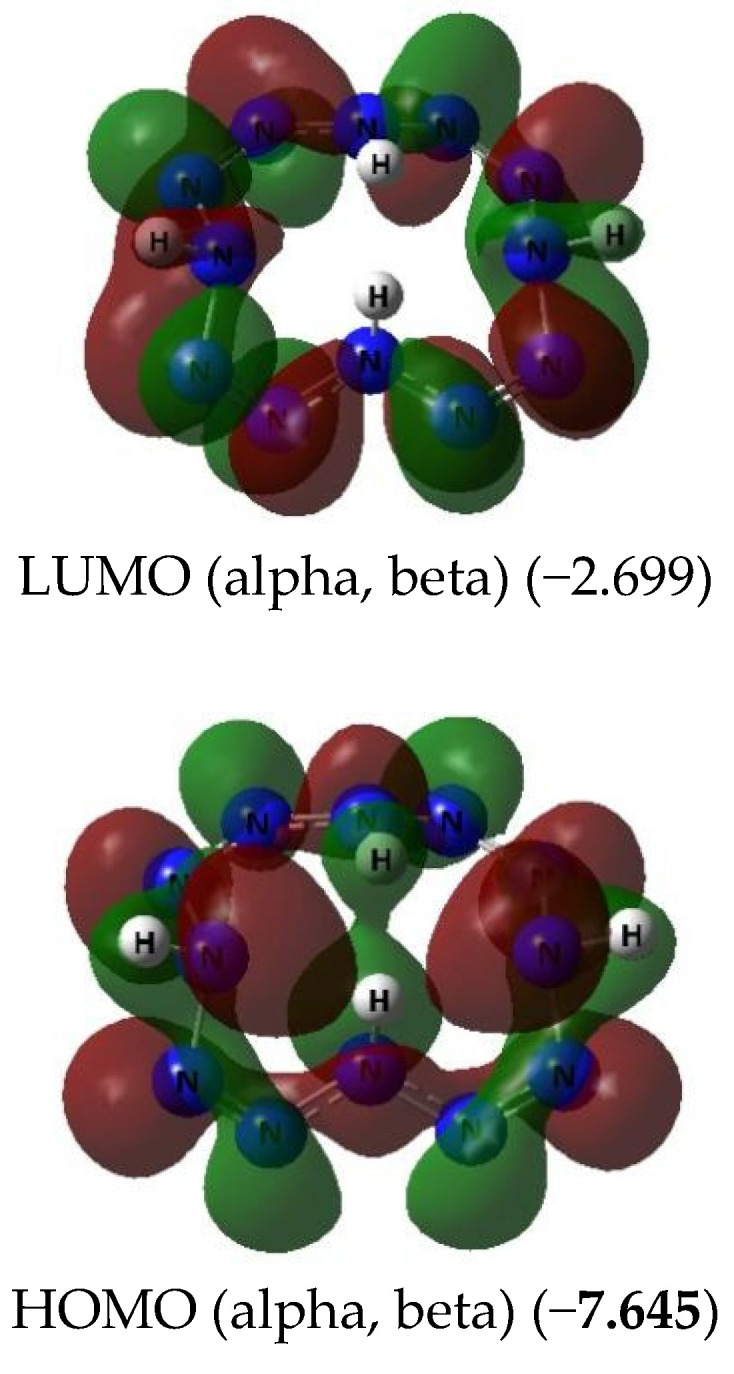
The images of highest occupied (HOMO) and lowest unoccupied (LUMO) molecular orbitals in the H_4_(N_12_) obtained by DFT M06/TZVP method. The values of energies of these molecular orbitals (in brackets) are given in eV.

**Figure 5:**
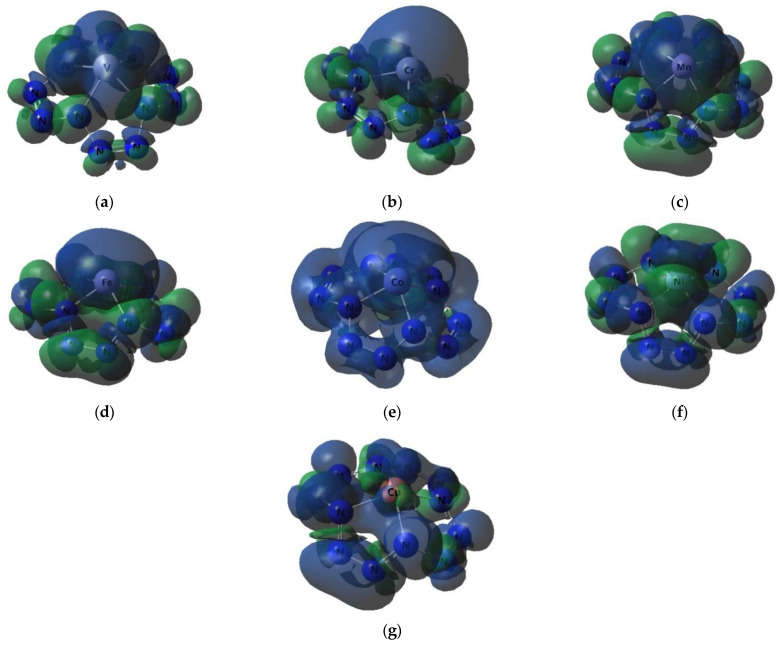
The images of spin density distribution in M(N_12_) complexes obtained by DFT M06/TZVP method: (**a**) V(N_12_), (**b**) Cr(N_12_), (**c**) Mn(N_12_), (**d**) Fe(N_12_), (**e**) Co(N_12_), (**f**) Ni(N_12_), (**g**) Cu(N_12_).

The images of the highest occupied and lowest vacant (unoccupied) molecular orbitals (HOMO and LUMO, respectively) produced by the DFT M06/TZVP method are presented in Figure 2, Figure 3 and Figure 4. These images demonstrated that there was no similarity between the shapes of both HOMO and LUMO for different M(N_12_) values. Interestingly, there was a marked difference in the energies of these MOs, not only for different complexes, but even for electrons with different spins in each of these complexes (Figure 2 and Figure 3). Spin density distribution diagrams of the studied complexes are shown in Figure 5.

Comparing the calculation data for the parameters of the molecular structures of the M(N_12_) compounds with the analogous parameters of the C(N_12_) compound described in our previous article [1], it can be noted that the M–N bond lengths for any of the above M are much larger than the bond length of C–N to C(N_12_). This is a quite expected result, since the radius of any of these M atoms was much larger than the radius of the carbon atom. At the same time, the lengths of the N–N bonds in these compounds did not differ much from each other, as a result of which the size of the cell formed by four nitrogen atoms N1, N4, N7 and N10 was almost the same. In the case of a carbon atom, the size of this cell turns out to be sufficient for the center of the atom to be in the plane of the [N1N4N7N10] atoms, while in the case of any of the M atoms, this size is insufficient. It is precisely because of this that in M(N_12_), the deviation of the M atoms from this plane takes place, which, on the whole, is more significant the greater the radius of the M(IV) ion.

The standard thermodynamic parameters of formation (*H*^0^_f,298_, *S*^0^_f,298_, and *G*^0^_f,298_) for the chemical compounds are presented in Table 4. All of these parameters were positive, and, therefore, these compounds could not be obtained from the most thermodynamically stable, simple substances formed by the corresponding 3*d*-element and nitrogen (i.e., 3*d*-metal and molecular dinitrogen N_2_). It is noteworthy that in most cases, *S*^0^_f,298_ values for M(N_12_) are higher than for H_4_(N_12_) (the only exception is V(N_12_), while for *G*^0^_f,298_, half of the M(N_12_) complexes (M = Cr, Co, Ni, Cu) have higher values of this parameter compared to those for the H_4_(N_12_), while the other half (M = Ti, V, Mn, Fe) have lower values (Table 4). The dynamics of changes in the values *H*^0^_f,298_ and *G*^0^_f,298_ in the series Ti-Cu are the same—when moving from Ti to Cr, they increase, from Cr to Mn they decrease, and from Mn to Cu they increase again, ultimately exceeding the corresponding values for H_4_(N_12_). Thus, it can be argued that for at least four of the eight M, owing to the formation of the M(N_12_) investigated compounds, stabilization of the N12 cyclic carcass s happens. In this connection, it should be noted that for the compound C(N_12_) similar in structural formula and spatial (molecular) structure as described in [1], the values of the parameters *H*^0^_f__,298_, *S*^0^_f__,298_ and *G*^0^_f__,298_, calculated by the method M06/TZVP, were 1793.1 kJ/mol, 377.7 J/mol∙K and 2020.7 kJ/mol respectively, i.e., quite close to those for M(N_12_) presented in Table 4.

In the end of this section of the article, it seems appropriate to compare the results of quantum chemical calculations of the molecular structures of these compounds obtained by the M06/TZVP method with the results of calculations of a higher level, in particular, by the DFT M062X/Def2TZVP method. Some of these data are presented in Table S14 (see Supplementary Materials). They are still incomplete (because the implementation of this method requires significantly more time and technological costs compared to those for the DFT M06/TZVP method), and so far, we have managed to obtain the corresponding data only for five compounds of the M(N_12_) type, namely for M = Cr, Mn, Fe, Co, Cu (which took about 2 months for us). As you can see when comparing them with similar data for the corresponding complexes of the above 3*d* elements obtained by the DFT M06/TZVP method, they are, on the whole, close to each other. Thus, it can be argued that the three versions of the DFT method we used, namely, those with the M06, B3PW91 OPBE functionals, and the TZVP basis set, are quite reliable for predicting the specifics of the molecular structures of the M(N_12_) compounds under consideration. 

Unfortunately, at this point in time, we could not use the simplest version of the MP method, namely MP2, to calculate any of the M(N_12_) compounds (due to problems with the convergence of the results and many times longer calculation time compared to even the DFT M062X/Def2TZVP method, we were unable to complete the calculation even within 8 (!!) months).

## 4. Conclusions

In this way, the data obtained using the DFT M06/TZVP method, unambiguously predicted the possible existence of new, so far unknown in chemical science, coordination compounds of various 3*d*-elements with nitrogen having M(N_12_) composition (M = Ti, V, Cr, Mn, Fe, Co, Ni, Cu). So, we also obtained similar results and conclusions using two other versions of the density functional theory, the DFT B3PW91/TZVP and DFT OPBE/TZVP methods, and also using the MP2 method (see Supplementary Materials, Table S14). However, due to significant time costs, it was possible to implement them only for four of the eight considered compounds M(N_12_), namely, for M = Cr, Fe, Co, Cu. 

Comparing the calculation data for the molecular structure parameters of the M(N_12_) compounds with the analogous parameters of the C(N_12_) compound described in our previous article [1], it can be noted that the M–N bond lengths for any of the above M were much larger than the bond length C–N to C(N_12_). This is a quite expected result, because the radius of any of these M atoms is much larger than the carbon atom radius. At the same time, the lengths of the N–N bonds in these compounds are not much different from each other, and as a result, the cell size formed by four nitrogen atoms N1, N4, N7 and N10 was almost the same. In the case of a carbon atom, the cell size that turns out to be sufficient for the atom center is in the plane of the [N1N4N7N10] atoms, while in the case of any M atoms, this size is insufficient. Because of this, in M(N_12_) there is an M atoms deviation from this plane, which, in general, is more significant the larger the ion M(IV) radius.

It is interesting and somewhat unexpected that, according to calculations, there is a division of M(N_12_) compounds into two categories, in the first of them (Ti(N_12_), V(N_12_), Cr(N_12_) and Co(N_12_)), all of the M–N bond lengths are the same; in the second (Mn(N_12_), Fe(N_12_), Ni(N_12_) and Cu(N_12_)), they are equal only in pairs. No correlation between the electronic configuration of the central atom 3*d* element M and the compound M(N_12_) belonging to the corresponding category was observed. The compound molecular structures in the first category have *C*_4*v*_ symmetry, and those in the second are completely asymmetric. It may be expected that the electric dipole moments of the first category of compounds would be lower compared to those of the second category. In fact, there is an inverse relationship between these values. The deviation from the MN_4_ chelate node coplanarity is also more pronounced in the first category of complexes. Nevertheless, the grouping of four N4 nitrogen atoms bonded to the M atom in any of these compounds is strictly planar. In this regard, the discussed compounds differed only in that in first category of compounds, all non-bonding angles between nitrogen atoms in this group were equal to 90.0°, while in second category of compounds, only pairwise equality of these angles, as well as the M–N bond lengths, was found.

In our opinion, the results of quantum chemical calculations within the framework of each of these three methods provide a reason for more thorough study of all 3*d*-metal-nitrogen-containing macrocyclic compounds analyzed in this article. It should be noted in this connection that the use of DFT computational methods of a higher level compared to DFT M06/TZVP (i.e., DFT M062X/Def2TZVP) gives practically the same results as the DFT M06/TZVP method (both in qualitative and quantitative terms), but requires less time. First of all, it is necessary to confirm their existence in the experiment. Judging by the very high values of ∆*H*^0^_f,298_ and ∆*G*^0^_f,298_ (more than 1500 kJ/mol), all of these compounds are high-energy substances, and that is why, it seems to us, if the synthesis of these exotic compound is successful, they will undoubtedly find some practical application, at least in the above capability.

## Figures and Tables

**Table 1:** Key parameters of molecular structures of M(N_12_) compounds calculated by DFT M06/TZVP method.

	3*d*-Element (M)								
Structural Parameter	Ti	V	Cr	Mn	Fe	Co	Ni	Cu	(H_4_N_12_)
M–N bond lengths in the MN_4_ chelate node, *pm*									
M1N1	193.5	188.2	186.3	191.1	183.7	181.2	179.6	186.0	-
M1N4	193.5	188.2	186.3	191.1	182.6	181.2	179.9	184.2	-
M1N7	193.5	188.2	186.3	190.0	182.6	181.2	179.6	184.2	-
M1N10	193.5	188.2	186.3	190.0	183.7	181.2	179.5	186.0	-
Nitrogen–nitrogen bond lengths in macrocycle, *pm*									
N1N2	138.0	137.9	139.1	131.7	145.3	137.4	140.6	146.3	148.1
N2N3	125.2	125.0	123.8	129.6	123.4	124.8	121.3	123.2	123.4
N3N4	138.0	137.9	139.1	131.7	137.2	137.4	151.2	134.5	135.1
N4N5	138.0	137.9	139.1	145.3	138.5	137.4	130.2	139.9	135.1
N5N6	125.2	125.0	123.8	123.7	124.3	124.8	131.4	122.5	123.4
N6N7	138.0	137.9	139.1	136.0	138.5	137.4	130.2	139.9	148.1
N7N8	138.0	137.9	139.1	138.8	137.2	137.4	151.2	134.5	149.5
N8N9	125.2	125.0	123.8	124.2	123.4	124.8	121.3	123.2	122.8
N9N10	138.0	137.9	139.1	138.7	145.3	137.4	140.6	146.3	136.1
N10N11	138.0	137.9	139.1	136.1	131.5	137.4	136.1	131.1	136.0
N11N12	125.2	125.0	123.8	123.7	130.0	124.8	125.6	129.8	122.8
N12N1	138.0	137.9	139.1	145.2	131.5	137.4	136.1	131.1	149.5
Distance from the center of the M atom to the plane formed by donor nitrogen atoms in the MN_4_ chelate node. *pm*									
	104.4	97.5	89.9	90.9	83.2	78.5	67.4	70.3	-
Bond angles in the MN_4_ chelate node, *deg*									
N1M1N4	73.1	74.4	76.6	77.9	77.8	79.2	81.9	81.8	-
N4M1N7	73.1	74.4	76.6	75.9	77.6	79.2	82.3	81.0	-
N7M1N10	73.1	74.4	76.6	75.5	77.8	79.2	81.9	81.8	-
N10M1N1	73.1	74.4	76.6	75.9	79.3	79.2	81.6	82.7	-
Bond angles sum (**BAS**), *deg*	**292.4**	**297.6**	**306.4**	**305.2**	**312.5**	**316.8**	**327.7**	**327.3**	-
Deviation from coplanarity, *deg*	**67.6**	**62.4**	**53.6**	**54.8**	**47.5**	**43.2**	**32.3**	**32.7**	-
Non-bond angles in the MN_4_ chelate node, *deg*									
N1N4N7	90.0	90.0	90.0	89.1	90.7	90.0	89.7	90.8	103.9
N4N7N10	90.0	90.0	90.0	90.9	90.7	90.0	89.7	90.8	68.1
N7N10N1	90.0	90.0	90.0	90.9	89.3	90.0	90.3	89.2	96.9
N10N1N4	90.0	90.0	90.0	89.1	89.3	90.0	90.3	89.2	68.1
Non-bond angles sum (NBAS), *deg*	360.0	360.0	360.0	360.0	360.0	360.0	360.0	360.0	337.0
Deviation from coplanarity, *deg*	0.0	0.0	0.0	0.0	0.0	0.0	0.0	0.0	23.0
Bond angles in 5-membered cycles, *deg*									
M1N1N2	119.7	119.8	118.6	116.1	117.0	117.4	116.5	113.0	-
N1N2N3	112.4	111.9	112.6	114.8	109.9	112.7	116.0	111.1	114.9
N2N3N4	112.4	111.9	112.6	114.8	114.4	112.7	110.1	119.2	116.7
N3N4M1	119.7	119.8	118.6	116.1	118.9	117.4	114.6	114.6	-
M1N4N5	119.7	119.8	118.6	116.6	118.6	117.4	113.4	114.9	-
N4N5N6	112.4	111.9	112.6	110.8	112.2	112.7	113.9	114.6	116.8
N5N6N7	112.4	111.9	112.6	115.5	112.2	112.7	113.9	114.6	114.9
N6N7M1	119.7	119.8	118.6	118.9	118.6	117.4	113.3	114.9	-
M1N7N8	119.7	119.8	118.6	118.5	118.9	117.4	114.6	114.6	-
N7N8N9	112.4	111.9	112.6	113.0	114.4	112.7	110.1	119.2	116.4
N8N9N10	112.4	111.9	112.6	113.0	109.9	112.7	116.0	111.0	120.9
N9N10M1	119.7	119.8	118.6	118.5	117.0	117.4	116.6	113.0	-
M1N10N11	119.7	119.8	118.6	118.9	116.8	117.4	115.3	111.3	-
N10N11N12	112.4	111.9	112.6	115.4	113.4	112.7	113.6	116.3	120.9
N11N12N1	112.4	111.9	112.6	110.8	113.4	112.7	113.6	116.3	116.4
N12N1M1	119.7	119.8	118.6	116.6	116.8	117.4	115.3	111.3	-
Deviation of macrocycle (N_12_) from coplanarity, *deg*	80.4	76.4	85.2	97.8	74.0	75.6	93.6	103.0	137.7
The difference between macrocycle deviation (N_12_) in (H_4_N_12_) and in M(N_12_), *deg*	57.3	61.3	52.5	39.9	63.7	52.1	44.1	34.7	-

**Table 2:** Electric dipole moments (μ, Debye) of M(N_12_) compounds calculated by DFT M06/TZVP method.

M	Ti	V	Cr	Mn	Fe	Co	Ni	Cu	(H_4_)
μ	8.43	7.30	4.81	5.40	4.65	4.00	2.92	3.11	4.75

**Table 3:** NBO analysis data for M(N_12_) and H_4_(N_12_) calculated by DFT M06/TZVP method.

Effective Charge on Atom, in Units of Electron Charge (ē)								<S**2>
M	M1	N1 (N4)	N2 (N6)	N3 (N5)	N7 (N10)	N8 (N12)	N9 (N11)	
Ti	1.1781	−0.3028	0.0042	0.0042	−0.3028	0.0041	0.0042	0
		(−0.3028)	−0.0042	−0.0042	(−0.3028)	−0.0041	−0.0042	
V	0.7623	−0.2071	0.0083	0.0083	−0.2071	0.0083	0.0083	0.7532
		(−0.2071)	−0.0083	−0.0083	(−0.2071)	−0.0083	−0.0083	
Cr	0.7069	−0.2275	0.0253	0.0254	−0.2275	0.0255	0.0254	2.2481
		(−0.2275)	−0.0253	−0.0254	(−0.2275)	−0.0255	−0.0254	
Mn	0.8408	−0.1900	0.0002	0.0002	−0.2975	0.0118	0.0121	3.9244
		(−0.1899)	−0.0629	(−0.0081)	(−0.2975)	(−0.0078)	−0.0629	
Fe	0.5588	−0.1263	0	0.0636	−0.2315	0.0636	0	2.0654
		(−0.2316)	−0.0102	−0.0101	(−0.1263)	−0.0045	−0.0045	
Co	0.5406	−0.1783	0.0216	0.0216	−0.1783	0.0216	0.0217	3.7832
		(−0.1783)	−0.0216	−0.0216	(−0.1783)	−0.0216	−0.0217	
Ni	0.4074	−0.1928	0.0748	0.0427	−0.1190	0.0427	0.0748	0.0364
		(−0.1190)	(−0.0096)	(−0.0095)	(−0.1928)	−0.0002	−0.0002	
Cu	0.6256	−0.1576	−0.0131	0.0729	−0.2579	0.073	−0.0131	0.7751
		(−0.2581)	−0.0361	−0.0361	(−0.1577)	−0.0068	−0.0729	
(H_4_)	-	−0.4932	−0.0262	0.0543	−0.4934	−0.0316	0.058	0
		(−0.3478)	(−0.0263)	−0.0544	(−0.3109)	(−0.0319)	−0.058	

The symbol (**) in this case means raising to the power of 2 (i.e., squaring).

**Table 4:** Standard thermodynamic parameters of formation (*H*^0^_f__,298_, *S*^0^_f__,298_ and *G*^0^_f__,298_) for various M(N_12_) calculated by DFT M06/TZVP method.

M	Standard Thermodynamic Parameters of Formation		
	*H*^0^_f,298_, kJ/mol	*S*^0^_f,298_, J/mol∙K	*G*^0^_f,298_, kJ/mol
Ti	1562.3	398.3	1792.8
V	1662.2	399.1	1890.4
Cr	1833.8	403.2	2061.7
Mn	1571.7	404.1	1801.0
Fe	1744.8	403.4	1972.9
Co	1823.6	404.2	2052.2
Ni	1933.7	412.6	2159.7
Cu	2033.9	405.0	2263.3
H4	1705.9	399.3	2004.8

## Data Availability

Not applicable.

## Supplementary Materials

The following supporting information can be downloaded at: https://www.mdpi.com/article/10.3390/ijms23126560/s1.
