# Supplementary material for: Twelve-Nitrogen-Atom Cyclic Structure Stabilized by 3d-Element Atoms: Quantum Chemical Modeling
Source: Int J Mol Sci. 2022 Jun 12;23(12):6560. doi: 10.3390/ijms23126560 (PMC9223744; doi:10.3390/ijms23126560)
Supplement: Supplementary file 1 [file ijms-23-06560-s001.zip › ijms-1741220-supplementary.pdf]

Table S1. Bond lengths and bond angles in the Ti(N_{12}) calculated by DFT B3PW91/TZVP, DFT OPBE/TZVP and DFT M06/TZVP methods.

Structural parameter	Calculated by		
	DFT B3PW91/TZVP	DFT OPBE/TZVP	DFT M06/TZVP
Titanium-nitrogen bond lengths in the TiN_4 node, *pm*			
Ti1N1	193.2	193.4	193.5
Ti1N4	193.2	193.3	193.5
Ti1N7	193.2	193.4	193.5
Ti1N10	193.2	193.3	193.5
Nitrogen-nitrogen bond lengths in macrocycle, *pm*			
N1N2	137.6	137.0	138.0
N2N3	125.7	127.2	125.2
N3N4	137.6	137.1	138.0
N4N5	137.6	137.1	138.0
N5N6	125.7	127.2	125.2
N6N7	137.6	137.0	138.0
N7N8	137.6	137.2	138.0
N8N9	125.7	127.2	125.2
N9N10	137.6	137.0	138.0
N10N11	137.6	137.0	138.0
N11N12	125.7	127.2	125.2
N12N1	137.6	137.2	138.0
Bond angles in the TiN_4 node, *deg*			
N1Ti1N4	73.1	72.6	73.1
N4Ti1N7	73.1	72.6	73.1
N7Ti1N10	73.1	72.6	73.1
N10Ti1N1	73.1	72.6	73.1
Bond angles sum (***BAS***), *deg*	**292.4**	**290.4**	**292.4**
Non-bond angles in the TiN_4 node, *deg*			
N1N4N7	90.0	90.1	90.0
N4N7N10	90.0	89.9	90.0
N7N10N1	90.0	90.1	90.0
N10N1N4	90.0	89.9	90.0
Non-bond angles sum (NBAS), *deg*	360.0	360.0	360.0

Bond angles in 5-membered cycles, *deg*			
Ti1N1N2	119.7	120.0	119.7
N1N2N3	112.3	111.8	112.4
N2N3N4	112.3	111.8	112.4
N3N4Ti1	119.7	120.0	119.7
Ti1N4N5	119.7	120.0	119.7
N4N5N6	112.3	111.8	112.4
N5N6N7	112.3	111.8	112.4
N6N7Ti1	119.7	120.0	119.7
Ti1N7N8	119.7	119.9	119.7
N7N8N9	112.3	111.8	112.4
N8N9N10	112.3	111.8	112.4
N9N10Ti1	119.7	120.0	119.7
Ti1N10N11	119.7	120.0	119.7
N10N11N12	112.3	111.8	112.4
N11N12N1	112.3	111.8	112.4
N12N1Ti1	119.7	119.9	119.7

Table S2. Bond lengths and bond angles in the $V(N_{12})$ calculated by DFT B3PW91/TZVP, DFT OPBE/TZVP and DFT M06/TZVP methods.

Structural parameter	Calculated by		
	DFT B3PW91/TZVP	DFT OPBE/TZVP	DFT M06/TZVP
Vanadium-nitrogen bond lengths in the VN_4 node, *pm*			
V1N1	188.0	187.7	188.2
V1N4	188.0	187.6	188.2
V1N7	188.0	187.7	188.2
V1N10	188.0	187.6	188.2
Nitrogen-nitrogen bond lengths in macrocycle, *pm*			
N1N2	137.3	137.0	137.9
N2N3	125.8	127.0	125.0
N3N4	137.3	137.1	137.9
N4N5	137.3	137.1	137.9
N5N6	125.8	127.0	125.0
N6N7	137.3	137.0	137.9
N7N8	137.3	137.2	137.9
N8N9	125.8	126.9	125.0
N9N10	137.3	137.0	137.9
N10N11	137.3	137.0	137.9
N11N12	125.8	126.9	125.0
N12N1	137.3	137.2	137.9

Bond angles in the VN_4 node, *deg*			
N1V1N4	74.4	74.0	74.4
N4V1N7	74.4	74.0	74.4
N7V1N10	74.4	74.1	74.4
N10V1N1	74.4	74.1	74.4
Bond angles sum (***BAS***), *deg*	**297.6**	**296.2**	**297.6**
Non-bond angles in the VN_4 node, *deg*			
N1N4N7	90.0	90.1	90.0
N4N7N10	90.0	89.9	90.0
N7N10N1	90.0	90.1	90.0
N10N1N4	90.0	89.9	90.0
Non-bond angles sum (NBAS), *deg*	360.0	360.0	360.0
Bond angles in 5-membered cycles, *deg*			
V1N1N2	119.8	120.1	119.8
N1N2N3	111.7	111.2	111.9
N2N3N4	111.7	111.1	111.9
N3N4V1	119.8	120.1	119.8
V1N4N5	119.8	120.1	119.8
N4N5N6	111.7	111.1	111.9
N5N6N7	111.7	111.2	111.9
N6N7V1	119.8	120.1	119.8
V1N7N8	119.8	120.0	119.8
N7N8N9	111.7	111.2	111.9
N8N9N10	111.7	111.2	111.9
N9N10V1	119.8	120.1	119.8
V1N10N11	119.8	120.1	119.8
N10N11N12	111.7	111.2	111.9
N11N12N1	111.7	111.2	111.9
N12N1V1	119.8	120.0	119.8

Table S3. Bond lengths and bond angles in the Cr(N_{12}) calculated by DFT B3PW91/TZVP, DFT OPBE/TZVP and DFT M06/TZVP methods.

Structural parameter	Calculated by		
	DFT B3PW91/TZVP	DFT OPBE/TZVP	DFT M06/TZVP
Chromium-nitrogen bond lengths in the CrN_4 node, *pm*			
Cr1N1	185.4	181.1	186.3
Cr1N4	185.4	181.1	186.3
Cr1N7	185.4	181.1	186.3
Cr1N10	185.4	181.1	186.3
Nitrogen-nitrogen bond lengths in macrocycle, *pm*			
N1N2	138.7	137.7	139.1
N2N3	124.3	126.6	123.8
N3N4	138.7	137.7	139.1
N4N5	138.7	137.7	139.1
N5N6	124.3	126.6	123.8
N6N7	138.7	137.7	139.1
N7N8	138.7	137.7	139.1
N8N9	124.3	126.6	123.8
N9N10	138.7	137.6	139.1
N10N11	138.7	137.6	139.1
N11N12	124.3	126.6	123.8
N12N1	138.7	137.7	139.1
Bond angles in the CrN_4 node, *deg*			
N1Cr1N4	76.5	75.7	76.6
N4Cr1N7	76.5	75.7	76.6
N7Cr1N10	76.5	75.7	76.6
N10Cr1N1	76.5	75.7	76.6
Bond angles sum (***BAS***), *deg*	**306.0**	**302.8**	**306.4**
Non-bond angles in the CrN_4 node, *deg*			
N1N4N7	90.0	90.0	90.0
N4N7N10	90.0	90.0	90.0
N7N10N1	90.0	90.0	90.0
N10N1N4	90.0	90.0	90.0
Non-bond angles sum (NBAS), *deg*	360.0	360.0	360.0

Bond angles in 5-membered cycles, *deg*			
Cr1N1N2	118.8	120.4	118.6
N1N2N3	112.3	110.3	112.6
N2N3N4	112.3	110.3	112.6
N3N4Cr1	118.8	120.4	118.6
Cr1N4N5	118.8	120.4	118.6
N4N5N6	112.3	110.3	112.6
N5N6N7	112.3	110.3	112.6
N6N7Cr1	118.8	120.4	118.6
Cr1N7N8	118.8	120.4	118.6
N7N8N9	112.3	110.3	112.6
N8N9N10	112.3	110.3	112.6
N9N10Cr1	118.8	120.4	118.6
Cr1N10N11	118.8	120.4	118.6
N10N11N12	112.3	110.3	112.6
N11N12N1	112.3	110.3	112.6
N12N1Cr1	118.8	120.4	118.6

Table S4. Bond lengths and bond angles in the Mn(N_{12}) calculated by DFT B3PW91/TZVP, DFT OPBE/TZVP and DFT M06/TZVP methods.

Structural parameter	Calculated by		
	DFT B3PW91/TZVP	DFT OPBE/TZVP	DFT M06/TZVP
Manganese-nitrogen bond lengths in the MnN_4 node, *pm*			
Mn1N1	189.3	178.8	191.1
Mn1N4	189.3	178.8	191.1
Mn1N7	188.4	178.8	190.0
Mn1N10	188.4	178.8	190.0
Nitrogen-nitrogen bond lengths in macrocycle, *pm*			
N1N2	132.3	138.7	131.7
N2N3	129.5	125.3	129.6
N3N4	132.3	138.7	131.7
N4N5	143.4	138.7	145.3
N5N6	124.4	125.3	123.7
N6N7	136.2	138.7	136.0
N7N8	138.2	138.7	138.8
N8N9	124.9	125.3	124.2
N9N10	138.1	138.8	138.7
N10N11	136.3	138.7	136.1
N11N12	124.4	125.3	123.7
N12N1	143.3	138.8	145.2

Bond angles in the MnN_4 node, *deg*			
N1Mn1N4	78.0	77.7	77.9
N4Mn1N7	76.1	77.7	75.9
N7Mn1N10	75.8	77.7	75.5
N10Mn1N1	76.1	77.7	75.9
Bond angles sum (***BAS***), *deg*	**306.0**	**310.8**	**305.2**
Non-bond angles in the MnN_4 node, *deg*			
N1N4N7	89.1	90.0	89.1
N4N7N10	90.9	90.0	90.9
N7N10N1	90.8	90.0	90.9
N10N1N4	89.2	90.0	89.1
Non-bond angles sum (NBAS), *deg*	360.0	360.0	360.0
Bond angles in 5-membered cycles, *deg*			
Mn1N1N2	116.7	119.5	116.1
N1N2N3	114.3	110.9	114.8
N2N3N4	114.3	110.9	114.8
N3N4Mn1	116.7	119.5	116.1
Mn1N4N5	116.9	119.5	116.6
N4N5N6	111.0	110.9	110.8
N5N6N7	114.5	110.9	115.5
N6N7Mn1	118.9	119.5	118.9
Mn1N7N8	118.5	119.5	118.5
N7N8N9	112.6	110.9	113.0
N8N9N10	112.7	110.9	113.0
N9N10Mn1	118.5	119.5	118.5
Mn1N10N11	118.8	119.5	118.9
N10N11N12	114.5	110.9	115.4
N11N12N1	111.1	110.9	110.8
N12N1Mn1	117.0	119.5	116.6

Table S5. Bond lengths and bond angles in the Fe(N_{12}) calculated by DFT B3PW91/TZVP, DFT OPBE/TZVP and DFT M06/TZVP methods.

Structural parameter	Calculated by		
	DFT B3PW91/TZVP	DFT OPBE/TZVP	DFT M06/TZVP
Iron-nitrogen bond lengths in the FeN_4 node, *pm*			
Fe1N1	181.6	179.1	183.7
Fe1N4	182.3	179.1	182.6
Fe1N7	182.3	179.1	182.6
Fe1N10	181.6	179.1	183.7
Nitrogen-nitrogen bond lengths in macrocycle, *pm*			
N1N2	137.4	141.2	145.3
N2N3	124.1	124.0	123.4
N3N4	143.3	141.1	137.2
N4N5	132.1	135.4	138.5
N5N6	129.9	127.7	124.3
N6N7	132.1	135.4	138.5
N7N8	143.3	141.1	137.2
N8N9	124.1	124.0	123.4
N9N10	137.4	141.2	145.3
N10N11	137.6	135.4	131.5
N11N12	125.3	127.8	130.0
N12N1	137.6	135.4	131.5
Bond angles in the FeN_4 node, *deg*			
N1Fe1N4	77.8	78.1	77.8
N4Fe1N7	79.5	79.1	77.6
N7Fe1N10	77.8	78.1	77.8
N10Fe1N1	77.8	79.1	79.3
Bond angles sum (***BAS***), *deg*	**312.9**	**314.4**	**312.5**
Non-bond angles in the FeN_4 node, *deg*			
N1N4N7	89.4	90.0	90.7
N4N7N10	89.4	90.0	90.7
N7N10N1	90.6	90.0	89.3
N10N1N4	90.6	90.0	89.3
Non-bond angles sum (NBAS), *deg*	360.0	360.0	360.0

Bond angles in 5-membered cycles, *deg*			
Fe1N1N2	118.8	118.5	117.0
N1N2N3	113.4	111.1	109.9
N2N3N4	110.3	111.1	114.4
N3N4Fe1	117.3	118.5	118.9
Fe1N4N5	117.3	118.7	118.6
N4N5N6	113.0	111.7	112.2
N5N6N7	113.0	111.7	112.2
N6N7Fe1	117.3	118.7	118.6
Fe1N7N8	117.3	118.5	118.9
N7N8N9	110.3	111.1	114.4
N8N9N10	113.4	111.1	109.9
N9N10Fe1	118.8	118.5	117.0
Fe1N10N11	118.7	118.7	116.8
N10N11N12	111.9	111.7	113.4
N11N12N1	111.9	111.7	113.4
N12N1Fe1	118.7	118.7	116.8

Table S6. Bond lengths and bond angles in the Co(N_{12}) calculated by DFT B3PW91/TZVP, DFT OPBE/TZVP and DFT M06/TZVP methods.

Structural parameter	Calculated by		
	DFT B3PW91/TZVP	DFT OPBE/TZVP	DFT M06/TZVP
Cobalt-nitrogen bond lengths in the CoN_4 node, *pm*			
Co1N1	180.9	180.0	181.2
Co1N4	180.9	180.0	181.2
Co1N7	180.9	180.0	181.2
Co1N10	180.9	180.0	181.2
Nitrogen-nitrogen bond lengths in macrocycle, *pm*			
N1N2	136.9	137.0	137.4
N2N3	125.7	126.5	124.8
N3N4	136.9	137.0	137.4
N4N5	136.9	137.0	137.4
N5N6	125.7	126.5	124.8
N6N7	136.9	137.0	137.4
N7N8	136.9	137.0	137.4
N8N9	125.7	126.5	124.8
N9N10	136.9	137.0	137.4
N10N11	136.9	137.0	137.4
N11N12	125.7	126.5	124.8
N12N1	136.9	137.0	137.4

Bond angles in the CoN_4 node, *deg*			
N1Co1N4	79.5	79.4	79.2
N4Co1N7	79.5	79.4	79.2
N7Co1N10	79.5	79.4	79.2
N10Co1N1	79.5	79.4	79.2
Bond angles sum (***BAS***), *deg*	**318.0**	**317.6**	**316.8**
Non-bond angles in the CoN_4 node, *deg*			
N1N4N7	90.0	90.0	90.0
N4N7N10	90.0	90.0	90.0
N7N10N1	90.0	90.0	90.0
N10N1N4	90.0	90.0	90.0
Non-bond angles sum (NBAS), *deg*	360.0	360.0	360.0
Bond angles in 5-membered cycles, *deg*			
Co1N1N2	117.3	117.7	117.4
N1N2N3	112.7	112.2	112.7
N2N3N4	112.7	112.2	112.7
N3N4Co1	117.3	117.7	117.4
Co1N4N5	117.3	117.7	117.4
N4N5N6	112.7	112.2	112.7
N5N6N7	112.7	112.2	112.7
N6N7Co1	117.3	117.7	117.4
Co1N7N8	117.3	117.7	117.4
N7N8N9	112.7	112.2	112.7
N8N9N10	112.7	112.2	112.7
N9N10Co1	117.3	117.7	117.4
Co1N10N11	117.3	117.7	117.4
N10N11N12	112.7	112.2	112.7
N11N12N1	112.7	112.2	112.7
N12N1Co1	117.3	117.7	117.4

Table S7. Bond lengths and bond angles in the Ni(N_{12}) calculated by DFT B3PW91/TZVP, DFT OPBE/TZVP and DFT M06/TZVP methods.

Structural parameter	Calculated by		
	DFT B3PW91/TZVP	DFT OPBE/TZVP	DFT M06/TZVP
Nickel-nitrogen bond lengths in the NiN_4 node, *pm*			
Ni1N1	180.1	180.0	179.6
Ni1N4	180.1	180.0	179.9
Ni1N7	180.1	180.0	179.6
Ni1N10	180.1	180.0	179.5
Nitrogen-nitrogen bond lengths in macrocycle, *pm*			
N1N2	139.6	139.0	140.6
N2N3	124.5	125.4	121.3
N3N4	139.6	139.0	151.2
N4N5	133.8	134.4	130.2
N5N6	128.6	128.9	131.4
N6N7	133.8	134.4	130.2
N7N8	139.6	139.0	151.2
N8N9	124.5	125.4	121.3
N9N10	139.6	139.0	140.6
N10N11	133.8	134.4	136.1
N11N12	128.6	128.9	125.6
N12N1	133.8	134.4	136.1
Bond angles in the NiN_4 node, *deg*			
N1Ni1N4	81.7	80.9	81.9
N4Ni1N7	81.7	81.4	82.3
N7Ni1N10	81.7	80.9	81.9
N10Ni1N1	81.7	81.4	81.6
Bond angles sum (***BAS***), *deg*	**326.8**	**324.6**	**327.7**
Non-bond angles in the NiN_4 node, *deg*			
N1N4N7	90.0	90.0	89.7
N4N7N10	90.0	90.0	89.7
N7N10N1	90.0	90.0	90.3
N10N1N4	90.0	90.0	90.3
Non-bond angles sum (NBAS), *deg*	360.0	360.0	360.0

Bond angles in 5-membered cycles, *deg*			
Ni1N1N2	115.5	116.2	116.5
N1N2N3	113.4	112.9	116.0
N2N3N4	113.4	112.9	110.1
N3N4Ni1	115.5	116.2	114.6
Ni1N4N5	115.5	116.1	113.4
N4N5N6	113.6	113.2	113.9
N5N6N7	113.6	113.2	113.9
N6N7Ni1	115.5	116.1	113.3
Ni1N7N8	115.5	116.2	114.6
N7N8N9	113.4	112.9	110.1
N8N9N10	113.4	112.9	116.0
N9N10Ni1	115.5	116.2	116.6
Ni1N10N11	115.5	116.1	115.3
N10N11N12	113.6	113.2	113.6
N11N12N1	113.6	113.2	113.6
N12N1Ni1	115.5	116.1	115.3

Table S8. Bond lengths and bond angles in the Cu(N_{12}) calculated by DFT B3PW91/TZVP, DFT OPBE/TZVP and DFT M06/TZVP methods.

Structural parameter	Calculated by		
	DFT B3PW91/TZVP	DFT OPBE/TZVP	DFT M06/TZVP
Copper-nitrogen bond lengths in the CuN_4 node, *pm*			
Cu1N1	185.2	183.7	186.0
Cu1N4	183.5	183.8	184.2
Cu1N7	183.5	183.7	184.2
Cu1N10	185.1	183.8	186.0
Nitrogen-nitrogen bond lengths in macrocycle, *pm*			
N1N2	144.1	136.3	146.3
N2N3	124.1	126.9	123.2
N3N4	134.9	136.1	134.5
N4N5	139.4	136.1	139.9
N5N6	123.2	126.9	122.5
N6N7	139.6	136.3	139.9
N7N8	134.7	136.0	134.5
N8N9	124.1	127.0	123.2
N9N10	144.4	136.1	146.3
N10N11	131.6	136.1	131.1
N11N12	129.9	127.0	129.8
N12N1	131.7	136.0	131.1

Bond angles in the CuN_4 node, *deg*			
N1Cu1N4	82.0	82.0	81.8
N4Cu1N7	81.3	82.0	81.0
N7Cu1N10	82.1	81.9	81.8
N10Cu1N1	82.9	81.0	82.7
Bond angles sum (***BAS***), *deg*	**328.3**	**327.8**	**327.3**
Non-bond angles in the CuN_4 node, *deg*			
N1N4N7	90.7	89.9	90.8
N4N7N10	90.7	90.1	90.8
N7N10N1	89.3	89.9	89.2
N10N1N4	89.3	90.1	89.2
Non-bond angles sum (NBAS), *deg*	360.0	360.0	360.0
Bond angles in 5-membered cycles, *deg*			
Cu1N1N2	113.3	114.3	113.0
N1N2N3	111.6	114.7	111.1
N2N3N4	118.3	114.9	119.2
N3N4Cu1	114.4	114.2	114.6
Cu1N4N5	114.8	114.2	114.9
N4N5N6	114.6	114.9	114.6
N5N6N7	114.5	114.7	114.6
N6N7Cu1	114.8	114.3	114.9
Cu1N7N8	114.4	114.3	114.6
N7N8N9	118.5	114.8	119.2
N8N9N10	111.5	114.7	111.0
N9N10Cu1	113.3	114.2	113.0
Cu1N10N11	112.0	114.2	111.3
N10N11N12	116.0	114.7	116.3
N11N12N1	115.9	114.8	116.3
N12N1Cu1	112.0	114.3	111.3

Table S9. Electric dipole moments (μ, Debye) of $M(N_{12})$ and $H_4(N_{12})$ compounds calculated by DFT B3PW91/TZVP and OPBE/TZVP methods.

M	Ti	V	Cr	Mn	Fe	Co	Ni	Cu	(H_4)
μ (B3PW91/ TZVP)	8.32	7.31	5.09	5.37	4.62	4.09	3.45	3.34	4.79
μ (OPBE/ TZVP)	8.05	7.00	5.93	4.08	3.83	3.72	3.35	3.46	4.46

Table S10. NBO analysis data for the $M(N_{12})$ and $H_4(N_{12})$ calculated by DFT B3PW91/TZVP and OPBE/TZVP methods.

DFT method	Effective charge on atom, in units of electron charge (ē)								
	M	M1	N1 (N4)	N2 (N6)	N3 (N5)	N7 (N10)	N8 (N12)	N9 (N11)	<S**2>
B3PW91/ TZVP	Ti	1.1228	-0.2836 (-0.2836)	0.0014 (0.0014)	0.0014 (0.0014)	-0.2836 (-0.2836)	0.0014 (0.0014)	0.0015 (0.0015)	0.0000
	V	0.7246	-0.1911 (-0.1911)	0.0049 (0.0049)	0.0050 (0.0050)	-0.1911 (-0.1911)	0.0049 (0.0049)	0.0050 (0.0050)	0.7529
	Cr	0.7033	-0.2184 (-0.2184)	0.0213 (0.0213)	0.0213 (0.0213)	-0.2184 (-0.2184)	0.0213 (0.0213)	0.0213 (0.0213)	2.1179
	Mn	0.7849	-0.1774 (-0.1771)	0.0018 (0.0569)	0.0020 (-0.0079)	-0.2737 (-0.2735)	0.0072 (-0.0073)	0.0073 (0.0569)	3.8415
	Fe	0.5025	-0.2052 (-0.1169)	0.0580 (0.0040)	0.0036 (0.0040)	-0.1170 (-0.2052)	0.0036 (0.0053)	0.0580 (0.0053)	2.4264
	Co	0.5263	-0.1694 (-0.1694)	0.0189 (0.0189)	0.0189 (0.0189)	-0.1694 (-0.1694)	0.0189 (0.0189)	0.0190 (0.0190)	3.7773
	Ni	0.4513	-0.1517 (-0.1517)	0.0388 (0.0001)	0.0388 (0.0001)	-0.1517 (-0.1517)	0.0388 (0.0001)	0.0388 (0.0001)	2.0002
	Cu	0.6419	-0.1599 (-0.2547)	-0.0155 (0.0314)	0.0678 (0.0319)	-0.2547 (-0.1600)	0.0680 (0.0097)	-0.0159 (0.0101)	0.7781
	(H_4)	-	-0.4944 (-0.3068)	-0.0327 (-0.0327)	0.0536 (0.0536)	-0.4944 (-0.3359)	-0.0291 (-0.0291)	0.0520 (0.0520)	0.0000
OPBE/ TZVP	Ti	1.0310	-0.2480 (-0.2480)	-0.0049 (-0.0049)	-0.0050 (-0.0050)	-0.2479 (-0.2479)	-0.0050 (-0.0050)	-0.0048 (-0.0048)	0.0000
	V	0.6359	-0.1601 (-0.1601)	0.0005 (0.0005)	0.0005 (0.0005)	-0.1601 (-0.1601)	0.0005 (0.0005)	0.0007 (0.0007)	0.7973
	Cr	0.3765	-0.1013 (-0.1013)	0.0035 (0.0036)	0.0036 (0.0035)	-0.1013 (-0.1010)	0.0035 (0.0035)	0.0035 (0.0035)	0.0000
	Mn	0.2198	-0.0961 (-0.0963)	0.0206 (0.0207)	0.0207 (0.0206)	-0.0963 (-0.0963)	0.0207 (0.0206)	0.0206 (0.0207)	0.7691
	Fe	0.3096	-0.1184 (-0.1188)	0.0416 (-0.0006)	0.0419 (-0.0006)	-0.1190 (-0.1182)	0.0420 (-0.0006)	0.0417 (-0.0007	2.0705
	Co	0.4290	-0.1421 (-0.1421)	0.0174 (0.0174)	0.0173 (0.0173)	-0.1421 (-0.1421)	0.0174 (0.0174)	0.0176 (0.0176)	3.7688
	Ni	0.4360	-0.1433 (-0.1433)	0.0338 (0.0005)	0.0338 (0.0005)	-0.1433 (-0.1433)	0.0338 (0.0005)	0.0338 (0.0005)	2.0075
	Cu	0.6437	-0.1795 (-0.2423)	0.0208 (0.0206)	0.0298 (0.0297)	-0.1795 (-0.2430)	0.0206 (0.0204)	0.0294 (0.0293)	0.7697
	(H_4)	-	-0.4934 (-0.2868)	-0.0284 (-0.0284)	0.0395 (0.0396)	-0.4931 (-0.3018)	-0.0288 (-0.0288)	0.0389 (0.0392)	0.0000

Table S11. The values of energies (are given in eV) of highest occupied (HOMO) and lowest unoccupied (LUMO) molecular orbitals, and values of gap. The symbol “a” corresponds to electron with spin (+1/2), “b”, to electron with spin (–l/2). Calculated using ***DFT M06/TZVP*** method.

	HOMO(a)	LUMO(a)	Δ (a)	HOMO(b)	LUMO(b)	Δ (b)
$Ti(N_{12})$	-7.959	-4.092	***3.868***	-7.959	-4.092	***3.868***
$V(N_{12})$	-8.119	-3.805	***4.315***	-8.123	-3.538	***4.585***
$Cr(N_{12})$	-7.046	-4.661	***2.385***	-8.401	-3.744	***4.656***
$Mn(N_{12})$	-7.306	-4.948	***2.358***	-8.138	-3.501	***4.637***
$Fe(N_{12})$	-7.525	-5.173	***2.353***	-8.297	-4.107	***4.190***
$Co(N_{12})$	-7.805	-3.381	***4.424***	-8.976	-5.161	***3.816***
$Ni(N_{12})$	-7.697	-5.206	***2.491***	-7.761	-5.154	***2.606***
$Cu(N_{12})$	-7.911	-4.355	***3.556***	-7.909	-5.314	***2.596***
$H_4(N_{12})$	-7.645	-2.699	***4.946***	-7.645	-2.699	***4.946***

Table S12. The values of energies (are given in eV) of highest occupied (HOMO) and lowest unoccupied (LUMO) molecular orbitals, and values of gap. The symbol “a” corresponds to electron with spin (+1/2), “b”, to electron with spin (–l/2). Calculated using ***DFT B3PW91/TZVP*** method.

	HOMO(a)	LUMO(a)	Δ (a)	HOMO(b)	LUMO(b)	Δ (b)
$Ti(N_{12})$	-7.807	-4.262	***3.545***	-7.807	-4.262	***3.545***
$V(N_{12})$	-8.067	-4.286	***3.781***	-7.975	-3.474	***4.501***
$Cr(N_{12})$	-6.945	-4.634	***2.311***	-8.204	-4.096	***4.108***
$Mn(N_{12})$	-7.136	-5.238	***1.898***	-7.999	-3.928	***4.071***
$Fe(N_{12})$	-7.314	-5.410	***1.904***	-8.351	-4.352	***3.999***
$Co(N_{12})$	-7.566	-3.541	***4.025***	-8.743	-5.428	***3.315***
$Ni(N_{12})$	-7.676	-3.601	***4.075***	-7.596	-5.617	***1.979***
$Cu(N_{12})$	-7.701	-4.590	***3.111***	-7.741	-5.610	***2.131***
$H_4(N_{12})$	-7.470	-3.040	***4.430***	-7.470	-3.040	***4.430***

Table S13. The values of energies (are given in eV) of highest occupied (HOMO) and lowest unoccupied (LUMO) molecular orbitals, and values of gap. The symbol “a” corresponds to electron with spin (+1/2), “b”, to electron with spin (–l/2). Calculated using ***DFT OPBE/TZVP*** method.

	HOMO(a)	LUMO(a)	Δ (a)	HOMO(b)	LUMO(b)	Δ (b)
$Ti(N_{12})$	-6.667	-4.924	***1.743***	-6.667	-4.924	***1.743***
$V(N_{12})$	-6.135	-4.356	***1.779***	-6.871	-4.038	***2.833***
$Cr(N_{12})$	-5.381	-4.570	***0.811***	-5.381	-4.570	***0.811***
$Mn(N_{12})$	-5.696	-5.026	***0.670***	-6.290	-4.662	***1.628***
$Fe(N_{12})$	-6.075	-5.578	***0.497***	-6.673	-5.003	***1.670***
$Co(N_{12})$	-6.320	-3.988	***2.332***	-7.528	-5.456	***2.072***
$Ni(N_{12})$	-6.400	-4.306	***2.094***	-6.200	-5.832	***0.368***
$Cu(N_{12})$	-6.501	-4.955	***1.546***	-6.668	-6.115	***0.553***
$H_4(N_{12})$	-6.278	-3.754	***2.524***	-6.278	-3.754	***2.524***

Table S14. Bond lengths and bond angles in the $Cr(N_{12})$, $Mn(N_{12})$, $Fe(N_{12})$, $Co(N_{12})$ and $Cu(N_{12})$ calculated by DFT M06-2X/Def2TZVP methods.

	3*d*-element (M)				
Structural parameter	Cr	Mn	Fe	Co	Cu
M–N bond lengths in the MN_4 chelate node, *pm*					
M1N1	193.4	196.0	189.4	184.5	188.3
M1N4	193.4	191.9	184.7	189.0	184.6
M1N7	193.4	191.9	184.7	189.0	184.6
M1N10	193.4	196.0	189.4	184.5	188.3
Nitrogen-nitrogen bond lengths in macrocycle, *pm*					
N1N2	132.3	130.7	144.1	136.4	145.5
N2N3	129.5	130.6	123.6	123.1	122.9
N3N4	132.3	130.7	136.6	145.0	135.4
N4N5	140.6	144.4	139.1	130.7	139.9
N5N6	123.8	123.6	123.6	130.5	122.2
N6N7	140.6	136.0	139.1	130.7	139.9
N7N8	132.3	139.0	136.6	145.0	135.4
N8N9	129.5	123.4	123.6	123.1	122.9
N9N10	132.3	139.0	144.1	136.4	144.5
N10N11	140.6	136.0	130.4	139.5	130.8
N11N12	123.8	123.6	131.3	122.8	130.4
N12N1	140.6	144.4	130.4	139.5	130.8
Bond angles in the MN_4 chelate node, *deg*					
N1M1N4	75.6	77.2	77.4	78.7	81.5
N4M1N7	74.9	75.3	77.4	79.8	80.7
N7M1N10	75.6	75.1	77.4	78.7	81.5
N10M1N1	74.9	75.3	78.6	78.5	82.6
Bond angles sum (***BAS***), *deg*	**301.0**	**303.9**	**310.8**	**315.7**	**326.3**
Deviation from coplanarity, *deg*	59.0	56.1	49.2	44.3	33.7
Non-bond angles in the MN_4 chelate node, *deg*					
N1N4N7	90.0	88.7	91.1	88.9	91.1
N4N7N10	90.0	91.3	91.1	88.9	91.1
N7N10N1	90.0	91.3	88.9	91.1	88.9
N10N1N4	90.0	88.7	88.9	91.1	88.9
Non-bond angles sum (NBAS), *deg*	360.0	360.0	360.0	360.0	360.0
Deviation from coplanarity, *deg*	0.0	0.0	0.0	0.0	0.0
Bond angles in 5-membered cycles, *deg*					
M1N1N2	118.2	115.2	115.9	117.6	112.8
N1N2N3	114.0	115.8	110.5	116.9	111.5
N2N3N4	114.0	115.8	115.8	110.8	119.2
N3N4M1	118.2	115.2	118.5	115.0	114.6
M1N4N5	117.2	115.9	118.2	114.1	114.9
N4N5N6	113.3	111.1	112.7	115.4	114.7
N5N6N7	113.3	116.6	112.7	115.4	114.7
N6N7M1	117.2	118.8	118.2	114.1	114.9
M1N7N8	118.2	118.3	118.5	115.0	114.6
N7N8N9	114.0	113.4	115.8	110.8	119.2
N8N9N10	114.0	113.4	110.5	116.9	111.5
N9N10M1	118.2	118.3	115.9	117.6	112.8
M1N10N11	117.2	118.8	115.8	117.2	110.5
N10N11N12	113.3	116.6	114.6	113.3	116.9
N11N12N1	113.3	111.1	114.6	113.3	116.9
N12N1M1	117.2	115.9	115.8	117.2	110.5
